# Determinant Factors of Anemia among Nonpregnant Women of Childbearing Age in Southwest Ethiopia: A Community Based Study

**DOI:** 10.1155/2014/391580

**Published:** 2014-11-16

**Authors:** Yaregal Asres, Tilahun Yemane, Lealem Gedefaw

**Affiliations:** Department of Medical Laboratory Science and Pathology, College of Public Health and Medical Sciences, Jimma University, Jimma, Ethiopia

## Abstract

*Background.* Anemia affects one-quarter of the world's population; nonpregnant women were one of the groups for whom it is common, making it a global public health problem. *Objective.* To determine prevalence and risk factors of anemia among nonpregnant women of childbearing age in Jimma town southwest Ethiopia. *Methodology.* We conducted a community based cross-sectional study involving 441 nonpregnant women. Data was collected over two months of period. We collected sociodemographic and related data using structured questionnaire. We collected four milliliters of venous blood and five grams of stool samples from each study participant for hematological and parasitological analysis. We performed statistical analysis using SPSS-V 16 software. *Result.* The prevalence of anemia was 16.1% (*n* = 71) with mean hemoglobin 12.96 g/dL (±1.04), among which 97.2% (*n* = 69) were mildly anemic. Age group of 25–36 years old, lower economic level, illiteracy, multiparity, having intestinal parasitic infection, using more than two sanitary pads per day during menstruation period, and low body mass index were found to be risk factors of anemia among the group. *Conclusion.* Prevalence of anemia indicates mild public health importance which shows it was indeed public health problem. Identified risk factors should be considered for prevention and control of anemia among the group.

## 1. Introduction

Anemia is a condition characterized by a decrease in the number of red blood cells (RBCs) and/or hemoglobin (Hb) concentration, resulting in a lower ability of blood to carry oxygen for physiologic needs [1]. Anemia in nonpregnant women (NPW) is considered severe when Hb concentration is <8 g/dL, moderate when it falls between 8 and 10.9 g/dL and mild when it falls between 11 and 11.9 g/dL [2, 3]. Anemia is a global health problem; when anemia prevalence is ≥40%, 20–39%, and 5–19%, it is considered as severe, moderate, and mild public health problem, respectively [1]. Anemia affects one-quarter of the world's population and NPW were one of the groups for whom it was common, making it a global public health problem [4]. Four hundred sixty-eight million (30.2%) NPW are anemic in the world; the highest prevalence is found in South-East Asia (35.7%), in Africa (47.5%) [4], and in Kumasi, Ghana (53.2%) [5], and in Ethiopia the prevalence of anemia ranges from 17% to 52.3% [4, 6, 7]. Moreover, reports indicated that anemia in NPW is a growing public health problem [8]. In this group of people, anemia has a number of consequences which includes reducing in working capacity and impaired immunity [9, 10]. It has also a direct impact during pregnancy, since 50% of anemic cases during pregnancy start at the time of conception [10]. Beginning pregnancy with depleted iron stores and/or low Hb concentration has been shown to increase risk of premature delivery, low birth weight, and fetal and maternal mortality [4, 9–13].

Anemia is multifactorial in its etiology that can be isolated but more often coexist which can be an indicator of both nutrition and health status [9]. Anemia is one of the most common nutritional deficiency disorders [14, 15]; the most significant contributor for the onset of anemia is iron deficiency [16]. Although nutritional anemia affects both sexes and all age groups, the problem is more prevalent among women [10]. In addition to nutritional deficiency, there are a number of risk factors including malaria, intestinal parasitic infections (IPI), chronic illness [7], economic status [17], and increased number of parity [18]. Moreover, NPW of childbearing age have an additional risk of developing anemia because of their periodic menstrual blood loss [18, 19] and a heavy menstrual period which could last more than five days [18, 20].

Anemia was considered as largely preventable and easily treatable if detected in time and strategies for its prevention and control are well documented [13, 20]. Despite these facts, still it has been continued to be a common cause of mortality and morbidity [13]. Suggested strategies aimed at prevention of anemia are focused on the major underlying causes and its risk factors [3, 21]. But data on relative contributions of the risk factors on anemia are limited in developing countries [3]. Available reports showed variations in anemia prevalence and associated risk factors on women of childbearing age which makes it difficult to effectively address the problem [3, 21]. The etiology of anemia in Ethiopia is not well established and the information available is limited particularly in nonpregnant women of child bearing age [21]. Therefore, this study was aimed at determining the prevalence of anemia and its risk factors among NPW of childbearing age in Jimma Town, Southwest Ethiopia.

## 2. Materials and Methods

### 2.1. Study Area and Population

The study was conducted in Jimma Town, urban area located 350 km Southwest of Addis Ababa, capital city of Ethiopia. It divided in to thirteen Kebeles (the smallest administrative units in Ethiopia). The total population of the town was one hundred thirty-four thousand and forty, of whom females account 49.7% [22]. It has two governmental hospitals and three health centers providing health care services. There were also thirteen health posts, one in each Kebele having more than one health extension worker in each health post. In addition to governmental health institutions, there were three private maternal health clinics in the town.

The sample size was determined using single population proportion general formula (*n* = *z*(1 − *α*/2)^2^ × *P*(1 − *p*)/*d* ^2^). We used 17% prevalence of anemia among the group [6], 5% marginal error, and 95% confidence level. Factor two for design effect and 10% anticipated nonresponse rate were considered. Final minimum sample size for the study was 477. We included NPW between 15 and 49 years old, who were permanent resident in the study area and voluntary to participate in the study while we excluded women who had been blood transfused within four months of data collection, who were on treatments for anemia and postpartum women less than a period of two weeks. Women who were not on current contraceptive and not confirmed for their pregnancy were requested for urine human chorionic gonadotropin test.

**Figure 1:**
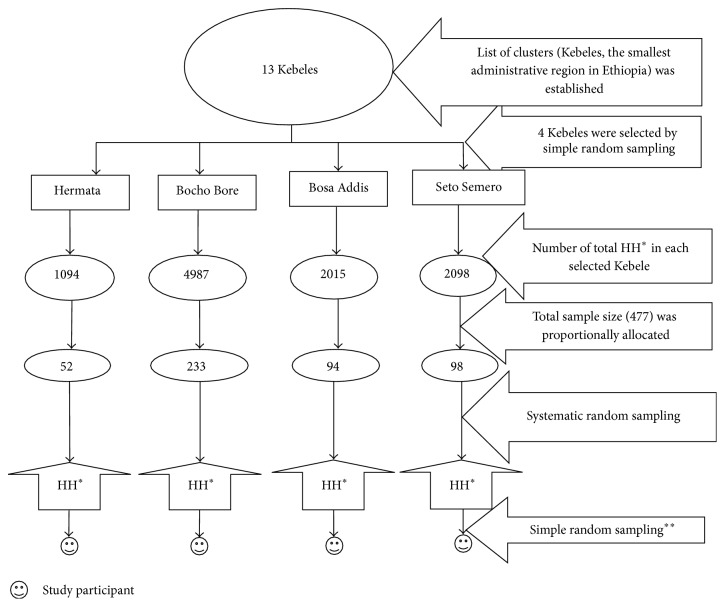
Sampling procedure in Jimma town, southwest Ethiopia, 2013.* NB*: HH^*^ = household; simple random sampling^**^ = simple random sampling technique was done when more than one of eligible NPW were found in a single household.

We used multistage sampling technique in a stepwise procedure to reach study participants. First, we selected four Kebeles by simple random sampling method considering that all Kebeles of the town were homogenous. The sum total household within four study Kebeles was ten thousand one hundred ninety-four. We allocated calculated sample size (477) to selected Kebeles proportional with the number of household in each Kebele (Figure 1). Then, proportionally allocated households, sampling units, were selected by systematic random sampling method using lists of the households as a sampling frame. Finally, we selected the study subjects by simple random sampling method from selected households if more than one of NPW was found in a single household. If eligible woman was not found in systematically selected house, the next household has been included.

Sociodemographic, socioeconomic, and clinical data were collected by trained clinical nurses using questionnaire based interview. Ethical approval was obtained from the Ethical Review Board of Jimma University (JU). We explained the objectives of the study for each study participant. We obtained written informed consent from 18–49-year-old study participants and from guardians of 15–18-year-old study participants. In addition, there was an assent section of the consent form for study participants aged 15–18 years old. Participants confirmed as anemic having an IPI and malaria were treated under consultation of physicians.

### 2.2. Blood Collection for Hematology Methods

Ethylenediaminetetraacetic acid (EDTA) anticoagulated venous blood sample (4 mL) was collected from each study participant using vacutainer system. Then the samples were transported to JU Hematology Laboratory within two hours of collection. We determined Hb concentration, mean corpuscular hemoglobin (MCH), mean corpuscular hemoglobin concentration (MCHC), and mean corpuscular volume (MCV) using CELL-DYN 1800 (Abbott, USA). We prepared both thick and thin blood films for assessment of hemoparasites and evaluation of red cell morphology.

### 2.3. Parasitological Survey

Stool sample (5 g) was collected from each study participant using clean, wide mouthed, and leak proof stool cups. Then, we examined stool samples in the nearby health center within 15 minutes of collection by wet mount preparation. Leftover samples were preserved using 10% formalin as a preservative and transported to JU Medical Parasitology Laboratory where we processed for formol-ether concentration technique. At the same day of blood and stool sample collection, we measured weight and height of NPW to compute their body mass index. Both thick and thin blood films were examined for the assessment of hemoparasites.

To ensure the quality of data, data collectors were trained and questionnaire was pretested. All laboratory activities were performed strictly following manufacturers' instruction and specific standard operating procedures. Quality control materials were used accordingly. All reagents and quality control materials were checked for their expiry date. Laboratory results were recorded on standard report formats according to unique identification number.

### 2.4. Statistical Analysis

We calculated body mass index (BMI) as weight in kilogram divided by the square of height in meter using Microsoft Office Excel 2007. We coded, cleaned, and entered all the data in to SPSS-V.16 statistical software for analysis. Descriptive statistics were used to give a clear picture of background information and determine prevalence of anemia. Binary logistic regressions were used to identify the candidate variables for multiple logistic regression analysis. All explanatory variables that have been associated with the outcome variable in bivariate analyses at 25% level of significance were considered as candidates to be entered into backward multiple logistic regression models. All variables with *P* value < 0.05 were considered as statistically significant.

### 2.5. Anemia

Women who had Hb value between 11–11.9 g/dL, 8–10.9 g/dL, and <8 g/dL were categorized as having mild, moderate, and severe anemia, respectively [2]. Normal values for MCHC (32–36 g/dL), MCH (27–33.5 pg) and MCV (80–100 fL) were used to classify anemia. Microcytosis was defined as MCV value less than 80 fL and hypochromia was defined as MCHC value less than 32 g/dL.

## 3. Results

### 3.1. Sociodemographic and Clinical Characteristics of Study Participants

Out of the total sample size (477), 441 NPW of childbearing age were enrolled in the study, with 92.45% response rate. Study participants were mainly within the age group of 25–35 years old with mean age of 28.9 ± 8.6 years. The household size of study participants ranged from 1 and 9 with an average of 4.3 persons per household. The majority of study participants were from household size of 1–5 (Table 1).

We checked all 441 study participants' blood and stool samples for hemoparasites and intestinal parasites, respectively. Accordingly, 33.3% (*n* = 147) had at least one intestinal parasite (Table 2). Single parasitic infection 93.2% (*n* = 137) was more prevalent. We identified a total of nine species of intestinal parasites among which* Ascaris lumbricoides* 37.6% (*n* = 59) took the highest proportion followed by* Giardia lamblia* 16.6% (*n* = 26),* Trichuris trichiura* 12.1% (*n* = 19),* Entamoeba histolytica*/*dispar* 10.8% (*n* = 17),* Hook worm* 8.3% (*n* = 13),* Strongyloides stercoralis* 4.5% (*n* = 7),* Schistosoma mansoni* 3.8% (*n* = 6),* Enterobius vermicularis* 3.8% (*n* = 6), and* Hymenolepis nana* 2.5% (*n* = 4).

### 3.2. Reproductive Health and Nutrition Related Characteristics of Study Participants

Among 264 contraceptive user study participants, 59.1% (*n* = 156) were used depo provera followed by pills, implant, and intrauterine device (IUD) with corresponding number of respondents 19.3% (*n* = 51), 18.2% (*n* = 48), and 3.4% (*n* = 9), respectively. Fifty-one (11.6%) study participants had BMI < 18.5 kg/m^2^ (Table 3).

### 3.3. Prevalence of Anemia among Study Participants

**Figure 2:**
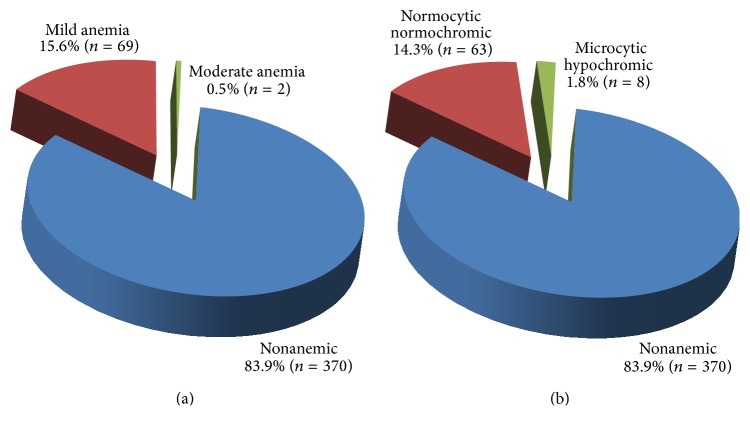
Severity (a) and morphological types (b) of anemia among nonpregnant women of childbearing age.* Mild anemia*: hemoglobin concentration between 11 g/dL and 11.9 g/dL;* moderate anemia*: hemoglobin concentration between 8 g/dL and 10.9 g/dL;* normocytic normochromic anemia*: hemoglobin concentration less than 12 g/dL, MCV value between 80 fL and 100 fL and MCHC value between 32 g/dL and 36 g/dL;* microcytic hypochromic anemia*: hemoglobin concentration less than 12 g/dL, MCV value less than 80 fL and MCHC value less than 32 g/dL.

The overall prevalence of anemia in this study was 16.1% (*n* = 71). Majority of anemic cases, 15.6% (*n* = 69), were mildly anemic and there was no identified severe anemia. The mean ± standard deviation results were 12.96 ± 1.04 g/dL for Hb concentration, 28.36 ± 1.60 pg for MCH, 32.15 ± 1.21 g/dL for MCHC, and 88.23 ± 4.5 fL for MCV. Most of the anemic cases, 14.3% (*n* = 63), had normocytic normochromic anemia (Figure 2).

### 3.4. Independent Predictors of Anemia among Study Participants

Thirteen explanatory candidate variables from binary logistic regression were entered into backward multiple logistic regression models. In the analysis, we found ten variables as predictors of anemia, and corresponding adjusted odds ratios were presented in Table 4.

## 4. Discussion

Random sampling procedures were followed to ensure that the selected study participants were representative of NPW of childbearing age population of the study area. Nearly one in six NPW of childbearing age (16.1%) was anemic in this study. According to the world health organization cutoff values [4], prevalence of anemia indicated mild public heath importance which showed it was indeed public health problem among the group in our study area.

The prevalence of anemia in this study is lower than another study in Ethiopia that reported 30.5% prevalence among NPW of childbearing age [7]. This might be due to the inclusion of rural villages in the study, which were not included in this study. Ethiopian policy of urban health extension program might have its own contribution to be so as the study was conducted during June-July 2005 before the program was implemented. The prevalence of anemia in this study is much lower compared to similar study done in another developing country, Bangladesh, reporting 73% prevalence [23]. The variation might be due to difference in study participants. The participants in Bangladesh are rural residents. Moreover, the study area was selected to represent areas covered only by government health services for women which did not have any additional health service by other providers while our study was done in urban areas and study population have an access to health service for both governmental and nongovernmental health institutions. This might also be a reason that severe anemia was not identified in our finding while similar study among NPW women in Bangladesh reported prevalence of mild, moderate, and severe anemia as 52%, 20%, and 1%, respectively [23]. The majority of the anemic cases in this study had normocytic normochromic anemia. This might be due to the reason that most determinant factors identified in this study were related to increased blood loss and/or red cell destruction resulting in normocytic normochromic anemia.

Our study finding indicated that participants with the age group of 25–35 years old were more likely to be anemic. Similar study in Egypt reported these age groups of NPW of childbearing age were relatively more affected than other age groups [26]. But reports in India indicated an age group of less than 25 years old were most affected [14, 18]. This variation might be due to the fact that in India half of child bearing age women had a birth before they were 20 years old and more than one in four before they were 18 years old; early childbearing was most common in India [24].

Lower economic level and nutritional status were significantly associated with anemia. NPW of childbearing age who had <500 Ethiopian birr (ETB) household income per month were more likely to be anemic compared to women who had >1,000 ETB. This could be due to the fact that those from lower economic status lack the ability to purchase the quality and/or quantity of foods compared with those from higher economic status. Similar studies in Ethiopia and Meghalaya [17, 18] supported this finding. Undernourished NPW of childbearing age who had a low BMI < 18.5 kg/m^2^ were 4.07 times more likely to have anemia compared to those who have BMI ≥ 18.5 kg/m^2^. This might be due to the fact that anemia is one of the most common nutritional deficiency disorders. It was supported by findings in India and Dar es Salaam, Tanzania [15, 25].

In our study, educational status and knowledge about anemia were significantly associated with anemia. Illiterate NPW of childbearing age and NPW who have not knowledge about anemia were more likely to be anemic compared with NPW who have knowledge about anemia and educated NPW up to secondary school and above. Similar studies in Ethiopia and Meghalaya [17, 21] supported this finding.

This study also showed that the proportion of anemia among NPW of childbearing age who had been infected with intestinal parasites was significantly higher, 3.05 more likely to be anemic, compared to those noninfected. It is in line with previous similar studies [17, 18]. This high prevalence of anemia among infected NPW might be explained by the fact that most identified intestinal parasites have their own contribution on blood loss and/or red cell destruction. The associations between anemia and chronic illness and malaria, which have been observed in similar studies [7], were not demonstrated in our study. This may be due to the low prevalence of malaria parasite infection identified in our study.

Those of NPW who were not using contraceptive were more likely to be anemic compared to contraceptive users. This might be due to the reason that 96.6% of users were using hormonal contraceptive methods. In addition to increase birth spacing between their children and reduce number of parity, hormonal contraceptives might have its own contribution on risk of menorrhagia. This finding was in line with similar reports in Ethiopia, Egypt, Dar es Salaam, Tanzania, and Bursa, Turkey [17, 25–27]. The positive contribution of contraceptive use and reduced number of children ever born were also providing supportive evidence in this regard.

Our study points out that using more than two sanitary pads per day during menstruation and history of bleeding other than delivery and menstruation were significantly associated with prevalence of anemia. These might be due to the fact that increasing number of sanitary pad usages per day reflects the amount of blood loss during menstruation which indicate heavy menstrual period. History of bleeding other than delivery and menstruation might have a consequence in additional blood loss among NPW of childbearing age.

## 5. Strengths and Limitations of the Study

Being a community based study and using primary data were the strengths of this study. On the other hand since we used a cross-sectional study design, it does not indicate which precedes first. Micronutrient (serum iron, folate, and vit-b_12_) levels, which might be root causes of anemia, were not assessed.

## 6. Conclusion

Anemia in this study is a mild public health problem; coordinated efforts should be paid to control anemia. Identified risk factors should be considered in prevention and control strategies of anemia among NPW of childbearing age in this region. Although predisposing factors for anemia were documented, large scale studies should be done to identify specific etiologies and root causes of anemia among the groups by assessing micronutrients (serum iron, folate, and vit-b_12_ levels).

## Figures and Tables

**Table 1:** Sociodemographic characteristics among nonpregnant women of childbearing age (*n* = 441).

Variable	Categories	Frequency (%)
Age	15–24	141 (32.0)
	25–35	203 (46.0)
	36–49	97 (22.0)

Marital status	Unmarried	83 (18.8)
	Married	358 (81.2)

Educational status	Illiterate	132 (30.0)
	Elementary^†^	160 (36.3)
	Secondary^‡^	149 (33.7)

Occupation	Employee	119 (27.0)
	Housewife	155 (35.1)
	Daily laborer	97 (22.0)
	Student	59 (13.4)
	Others	11 (2.5)

Household size	1–5	350 (79.4)
	>5	91 (20.6)

Monthly household income	<500	63 (14.3)
	500–1000	162 (36.7)
	>1000	216 (49.0 )

Elementary^†^ = elementary school (1–8 years of education), secondary^‡^ = secondary school and above (>8 years of education).

**Table 2:** Association of anemia with sociodemographic and clinical factors among nonpregnant women of childbearing age (*n* = 441).

Variable	Categories	Anemia		COR (95% CI)	*P* values
		Yes (%)	No (%)		
Age	15–24	21 (14.9)	120 (85.1)	1.52 (0.68, 3.39)	0.304
	25–35	40 (19.7)	163 (80.3)	2.14 (1.02, 4.48)	0.045
	36–49	10 (10.3)	87 (89.7)	1	0.111

Marital status	Unmarried	6 (7.2)	77 (92.8)	1	<0.001
	Married	65 (18.2)	293 (81.8)	2.85 (2.19, 6.82)	0.019

Educational status	Illiterate	34 (25.8)	98 (74.2)	2.69 (1.42, 5.1)	0.002
	Elementary^†^	20 (12.5)	140 (87.5)	1.11 (0.56, 2.21)	0.768
	Secondary^‡^	17 (11.4)	132 (88.6)	1	0.002

Occupation	Employee	12 (10.0)	107 (90.0)	1	0.001
	Housewife	22 (14.2)	133 (85.8)	1.48 (0.74, 3.12)	0.309
	Daily laborer	28 (28.9)	69 (71.1)	3.62 (1.73, 7.59)	0.001
	Student	5 (8.5)	54 (91.5)	0.83 (0.28, 2.46)	0.731
	Others	4 (36.4)	7 (63.6)	5.1 (1.32, 19.67)	0.019

Household size	1–5	56 (16.0)	294 (84.0)	1	
	>5	15 (16.5)	76 (83.5)	1.04 (0.56, 1.93)	0.911

Household income^*^	<500	23 (36.5)	40 (63.5)	14.9 (6.25, 35.78)	<0.001
	500–1000	40 (24.7)	122 (75.3)	8.53 (3.86, 18.80)	<0.001
	>1000	8 (3.7)	208 (96.3)	1	<0.001

Chronic illness	Yes	7 (70.0)	3 (30.0)	13.38 (3.37, 35.1)	0.026
	No	64 (14.8)	367 (85.2)	1	<0.001

History of bleeding^*^	Yes	16 (35.6)	29 (64.4)	3.42 (1.74, 6.71)	<0.001
	No	55 (13.9)	341 (86.1)	1	

Ever heard about anemia	Yes	15 (8.4)	164 (91.6)	1	
	No	56 (21.4)	206 (78.6)	2.97 (1.62, 5.45)	<0.001

Presence of current malaria	Yes	3 (60.0)	2 (40.0)	8.12 (1.33, 49.49)	0.023
	No	69 (15.8)	367 (84.5)	1	

Presence of current IPI	Yes	45 (30.6)	102 (69.4)	4.55 (2.67, 7.76)	<0.001
	No	26 (8.8)	268 (91.2)	1	<0.001

COR = crude odds ratio, CI = confidence interval, IPI = intestinal parasitic infection, household income^*^ = household income per month in Ethiopian birr, history of bleeding^*^ = history of bleeding other than menstruation and delivery (accidents, ulcer, and hemorrhoids), elementary^†^ = elementary school (1–8 years of education), and secondary^‡^ = secondary school and above (>8 years of education).

**Table 3:** Association of anemia with reproductive health and nutrition related factors among nonpregnant women of childbearing age.

Variable	Categories	Anemia		COR (95% CI)	*P* values
		Yes (%)	No (%)		
Parity	No	6 (5.5)	104 (94.5)	1	0.013
	1-2	29 (18.8)	125 (81.1)	4.02 (1.60, 10.06)	0.003
	3–5	31 (20.4)	121 (79.9)	4.44 (1.78, 11.06)	0.001
	>5	5 (20.0)	20 (80.0)	4.33 (1.21, 15.58)	0.025

Place of delivery service^*^	Yes	27 (13.0)	181 (87.0)	1	
	No	38 (30.9)	85 (69.1)	2.99 (1.72, 5.23)	<0.001

State of current breast feeding	Yes	28 (25.9)	80 (74.1)	1.76 (1.01, 3.07)	0.047
	No	37 (16.6)	186 (83.4)	1	

Birth spacing^**^	≤2	39 (33.1)	79 (66.9)	5.31 (2.62, 10.74)	<0.001
	>2	12 (8.5)	129 (91.5)	1	

Current contraceptive use	Yes	34 (12.9)	230 (87.1)	1	
	No	37 (20.9)	140 (79.1)	1.79 (1.07, 2.98)	0.026

Regularity of menstrual cycle	Regular	51 (18.3)	227 (81.7)	1.61 (0.92, 2.81)	0.092
	Irregular	20 (12.3)	143 (87.7)	1	

Length of blood flow in each menses	1–5	52 (13.4)	335 (86.7)	1	
	≥6	19 (35.2)	35 (64.8)	3.5 (1.86, 6.57)	<0.001

Sanitary pad usage per day	1-2	38 (11.4)	296 (88.6)	1	
	≥3	33 (30.8)	74 (69.2)	3.47 (2.04, 5.91)	<0.001

BMI in kg/m^2^	<18.5	24 (47.1)	27 (52.9)	6.49 (3.45, 12.16)	<0.001
	≥18.5	47 (12.5)	343 (87.5)	1	

COR = crude odds ratio, CI = confidence interval, BMI = body mass index, place of delivery service^*^ = place of delivery service in health institution during the recent birth, and birth spacing^**^ = birth spacing between the recent two consecutive children.

**Table 4:** Independent predictors of anemia from multivariate logistic regression model among nonpregnant women of childbearing age (*n* = 441).

Variable	Categories	AOR (95% CI)	*P* value
Age in year	15–24	2.42 (0.92, 6.37)	0.073
	25–35	6.53 (1.82, 13.39)	0.004
	36–49	1	0.016

Educational status	Illiterate	2.16 (1.67, 5.18)	0.043
	Elementary^†^	1.4 (.15, 1.64)	0.061
	Secondary^‡^	1	0.005

Household income^*^	<500	8.84 (6.47, 14.91)	<0.001
	500–1000	5.91 (2.31, 13.09)	<0.001
	>1000	1	<0.001

History of bleeding^**^	Yes	3.28 (1.24, 8.68)	0.017
	No	1	

Ever heard about anemia	Yes	1	
	No	3.33 (1.5, 7.42)	0. 003

IPI	Yes	3.34 (1.66, 6.73)	0.001
	No	1	

Children ever born	No child	1	<0.001
	1-2	7.73 (4.53, 16.93)	<0.001
	3–5	14.93 (10.58, 23.09)	<0.001
	>5	13.37 (4.83, 23.52)	<0.001

Current contraceptive use	Yes	1	
	No	4.77 (2.19, 10,42)	<0.001

Sanitary pad usage per day	1-2	1	
	≥3	3.03 (1.43, 6.41)	0.004

BMI in kg/m^2^	<18.5	4.07 (1.69, 9.84)	0.002
	≥18.5	1	

AOR = adjusted odds ratio; CI = confidence interval; BMI = body mass index; household income^*^ = household income per month in Ethiopian birr; history of bleeding^**^ = history of bleeding other than menstruation and delivery (accident, ulcer, and hemorrhoids); elementary^†^ = elementary school (1–8 years of education); secondary^‡^ = secondary school and above (>8 years of education).
